# Epidemiology of HEV Infection in Blood Donors in Southern Switzerland

**DOI:** 10.3390/microorganisms11102375

**Published:** 2023-09-22

**Authors:** Stefano Fontana, Paolo Ripellino, Christoph Niederhauser, Nadja Widmer, Peter Gowland, Orlando Petrini, Manuela Aprile, Giorgio Merlani, Florian Bihl

**Affiliations:** 1 Servizio Trasfusionale CRS della Svizzera Italiana, 6900 Lugano, Switzerland; manuela.aprile@trasfusionale.ch; 2 Blood Transfusion Unit, Faculty of Biology and Medicine, University of Lausanne, 1015 Lausanne, Switzerland; 3 Department of Neurology, Neurocenter of Southern Switzerland EOC, 6900 Lugano, Switzerland; paolo.ripellino@eoc.ch; 4 Faculty of Biomedical Sciences, Università della Svizzera Italiana, 6900 Lugano, Switzerland; 5 Interregional Blood Transfusion SRC, 3008 Berne, Switzerlandnadja.widmer@itransfusion.ch (N.W.); peter.gowland@itransfusion.ch (P.G.); 6 Institute for Infectious Diseases, University of Berne, 3008 Berne, Switzerland; 7 Institute of Microbiology, University of Applied Sciences and Arts of Southern Switzerland, 6500 Bellinzona, Switzerland; orlando@poleconsult.com; 8 Chief Medical Officer Office, Division of Public Health, Department for Health and Social Affairs, 6500 Bellinzona, Switzerland; giorgio.merlani@ti.ch; 9 Epatocentro Ticino, Via Soldino 5, 6900 Lugano, Switzerland; florian.bihl@gastromedical.ch; 10 Division of Gastroenterology and Hepatology, University Hospital Geneva, 1200 Geneva, Switzerland

**Keywords:** hepatitis E, seroprevalence, NAT testing, blood donors, transfusion safety

## Abstract

From 2014 to 2016, the number of hepatitis E virus (HEV) infections in southern Switzerland increased dramatically and suggested food as a potential infection reservoir. We evaluated the effects of food control measures introduced to limit HEV infections, assessing anti-HEV IgG and IgM rates in blood donors before and after the implementation of food control measures in 2017. From 2012 to 2013, we screened 1283, and from 2017 to 2019, we screened 1447 donors for IgG and IgM antibodies. No statistically significant differences were detected for IgG (32.8% from 2012 to 2013 vs. 31.1% from 2017 to 2019, *p* = 0.337) or IgM rates (2.0% from 2012 to 2013 vs. 2.8% from 2017 to 2019, *p* = 0.21). Rural provenience and age > 66 are predictors for positive IgG serology. A total of 5.9% of 303 donors included in both groups lost IgG positivity. We also determined nucleic acid testing (NAT) rates after the introduction of this test in 2018, comparing 49,345 donation results from southern Switzerland with those of 625,559 Swiss donor controls, and only 9 NAT-positive donors were found from 2018 to 2023. The high HEV seroprevalence in southern Switzerland may depend on different food supply chains in rural and urban areas. Local preventive measures probably have a limited impact on blood HEV risk; thus, continuous NAT testing is recommended.

## 1. Introduction

Hepatitis E virus (HEV) is a frequent cause of acute viral hepatitis [1,2]. Four different genotypes are known to cause infections in humans, genotype 3 being the main source in industrialized countries, including Europe [1,2].

IgG prevalence rates show that a high proportion of the population in European countries had contact with the virus, although at highly variable regional rates [3]. The genotype 3 HEV infection usually follows an asymptomatic course; therefore, only in the last few years was it recognized as a clinically relevant source of hepatitis E [4,5]. Iatrogenic transmission among humans through infected blood and blood products, however, has also been documented [4,6].

HEV can cause acute hepatitis, acute liver failure or acute-on-chronic liver failure, mostly in immunosuppressed patients such as transplant recipients, and chronic liver disease up to end-stage liver disease in immunosuppressed patients [7,8,9]. Extrahepatic manifestations of HEV infections are frequent; for instance, neurological manifestations can be severe and heavily impact morbidity [10,11,12]. In acute symptomatic HEV, about 10% of cases may develop neuralgic amyotrophy [13,14].

During primary infection, HEV causes a viremia that may last several weeks [7,15]. This, combined with the limited extent of symptoms in healthy individuals, makes this virus potentially transmissible by blood. Because of the potentially large diffusion in the population, acute HEV infection is thus relevant for transfusion safety [16]. So far, more than 50 transfusion-transmitted cases have been confirmed by molecular methods [11], but the numbers are probably much higher [17]. For this reason, several countries have recently introduced systematic blood donor screening by HEV nucleic acid testing (NAT) [18,19].

In 2014, reports of HEV infections in southern Switzerland rapidly increased, and between 2014 and 2017, a regional outbreak was observed [20]. Among the affected patients, several cases with moderate to severe liver disease and numerous cases with neurological complications were reported [21,22]. A Switzerland-wide study performed on blood donors between 2014 and 2016 showed an overall anti-HEV IgG seroprevalence of 20.4%, with the highest value of 33.6% observed in southern Switzerland [23]. This last observation, however, was performed in a small number (345) of blood donors and is not necessarily representative of the region.

The consumption of HEV-contaminated raw pork meat seems to be an important source of transmission to humans [24,25,26,27]. In fact, pigs are considered the main asymptomatic reservoir [4], with varying regional seroprevalence rates [28]. In Switzerland, for instance, the estimated overall seroprevalence in pigs is approximately 60% [29,30]. Numerous studies have shown that meat products from domestic pigs, especially pork sausages containing raw liver, are contaminated with HEV [31,32]. In southern Switzerland, the “mortadella di fegato crudo” is a traditional sausage consumed by a large part of the population, especially in rural zones. It contains raw pork liver, and it has been known since the 17th century [33]. The increasing number of clinical cases and the high IgG prevalence raised some health concerns in the local health authorities. Because the control of the food chain may be a cost-effective method to ensure blood safety [21], since 2017, raw pork liver can no longer be used for the production of “mortadella di fegato crudo” or other liver-containing sausages.

Triggered by similar concerns, the increasing international awareness for HEV [17], and national seroepidemiological data from blood donors [23], the Swiss Blood Transfusion Service of the Swiss Red Cross (BTS SRC) decided to introduce mandatory HEV NAT screening for all blood donations starting on 1 October 2018. In southern Switzerland, HEV NAT screening started already on 17 September 2018.

This study was carried out to assess the impact of food control measures introduced in 2017 for HEV prevention in Switzerland. We analyzed the HEV IgM and IgG seroprevalence in the southern Swiss donor population before and after the local HEV outbreak and the introduction of food control measures in 2017, with the aim to assess the IgG and IgM HEV seroprevalence in blood donors of southern Switzerland in the 2017–2019 period, shortly after the introduction of the food control measures. Further, we compared the seroprevalence from 2017 to 2019 with that of the 2012–2013 period, before the HEV outbreak, and analyzed the regional IgG and IgM distribution for differences between urban and rural areas. We also investigated changes in the antibody status of IgG in a subgroup of donors included in both groups 2012–2013 and 2017–2019 and evaluated the effects on blood donation safety by the systematic HEV NAT screening introduced in 2017 in Switzerland.

## 2. Materials and Methods

The study protocol was approved by the local ethics committee and was conducted in accordance with the Declaration of Helsinki. All participants signed the general consent for research for blood donors of BTS SRC. The study was registered at clinicaltrial.gov with the number NCT03601221.

### 2.1. Geographic Area Studied and Food Chain Control Measures

Canton Ticino in southern Switzerland is characterized by a gradient from the rather urban southern area, a hilly region with several small cities, agglomerations, industries, and services, and the almost exclusively rural northern section, a typical alpine region with small villages and farms. The canton is divided into eight administrative districts, from which we collected data. We also included the Italian part of Canton Grison, which belongs to Canton Grisons but is located southernly of the Alps, is included in our blood donation region, and shares the same alimentary habits as Ticino. We then defined three different areas to be compared: the south (urban), the center (mixed urban–rural), and the north (rural). The south corresponds to the urban districts of Lugano and Mendrisio, the center to the Districts of Locarno and Bellinzona, and the north to the prevalently rural districts of Blenio, Leventina, Riviera, and Vallemaggia. Grigioni Italiano was included in the latter area.

Based on the Federal Act for Food Safety and its Ordinance, health authorities of Canton Ticino no longer allow the use of pork raw liver in foodstuff, enforcing the new legal frame as of April 2017. The most important changes were the obligation to cook any food containing pork liver for at least 20 min at 71 °C; the selection and qualification of the providers of pork raw liver by product testing; testing the pork liver used for the manufacture of food products by HEV RNA molecular methods; replacing pork liver with liver from other animal species; and the release of the finished food products only after HEV RNA testing. Additionally, in January 2018 and based on a request by the Cantonal Chief Medical Officer, the Federal Office of Public Health added hepatitis E to the list of infectious diseases requiring compulsory notification, as set out in the Federal Act on Controlling Communicable Human Diseases (“Epidemics Act”).

### 2.2. HEV IgG and IgM Seroprevalence and Regional Distribution

Between May 2017 and April 2019, blood donations from 1447 donors from urban and rural regions were prospectively screened for anti-HEV IgG and IgM antibodies (Wantai ELISA, Eurobio, Les Ulis, France). The results are presented as ratios of optical density (OD) divided by the cut-off OD, a ratio over 1.00 being considered positive. The donors were recruited according to their attendance at three blood donation centers situated in Lugano, Bellinzona, and Locarno, as well as during mobile blood donation collections in little villages of the other districts. The number of donors per district was set arbitrarily and proportionally according to their population. In small districts, 79 samples were obtained from May to December 2017 to allow the collection of enough samples. All donors were 18–75 years old and fulfilled the blood donation requirements of the BTS SRC.

Samples were collected from each donor before starting the blood donation using 10 mL EDTA tubes used for routine laboratory testing of infectious diseases (BD, Eysins, Switzerland), immediately transported to the laboratory, and centrifuged at room temperature. After routine testing, the plasma was frozen at −25 °C until analysis.

### 2.3. Course of the HEV Seroprevalence between 2012–2013 and 2017–2019

To evaluate whether the observed increase in symptomatic HEV cases in southern Switzerland was real or only related to an increasing awareness toward HEV, we compared the IgG seropositivity frequency of the 2017–2019 samples with that of 1283 frozen donor archive plasma samples collected between May 2012 and April 2013. No comparison was possible for the IgM seropositivity because the analyses of these samples were performed retrospectively. Plasma samples of the 2012–2013 period were from donors selected to fulfill the same criteria as the 2017–2019 donors, i.e., the distribution was based on the population of every district, with a slight overrepresentation of the rural regions, and donations had been performed in the three centers Lugano, Locarno, and Bellinzona or collected during mobile donation sessions in the districts included in the 2017–2018 period. Sessions were chosen retrospectively based on the mobile donation collection plans of 2012 and 2013. For both collection periods, only age and sex but no additional demographic or medical data were available.

Because the HEV viremia in infected but asymptomatic donors is limited over time, and the inclusion of two donations (usually at least 6 months apart) by the same donor has virtually no influence on the significance of the results, we accepted the risk that samples could be collected from the same donor during two different donations within the 2012–2013 time frame.

Donors included in both the 2012–2013 and 2017–2019 study periods were identified, and potential changes in their IgG seropositivity from the first to the second period were assessed. We calculated the percentage of donors with IgG changes (acquisition or loss) and of those with unchanged results. In a few cases, when the same donor donated twice in the period 2012–2013, the last 2012–2013 donation was used for this analysis.

### 2.4. HEV NAT Incidence 2018–2023

During the 4.5 years covering the period from 17 September 2018 to 16 March 2023, 49,345 blood donations were tested for HEV NAT in southern Switzerland using the Procleix^®^ HEV Assay (Grifols, Barcelona, Spain), a test with a 95% lower level of sensitivity of 7.89 IU/mL. Whole blood donations were analyzed in pools of 12 samples or less and apheresis donations as individual samples. For this test, the BTS SRC requires a minimal sensitivity of 450 IU/mL. Positive pools were reanalyzed to validate the measurement, and if the second analysis was also positive, all individual samples were tested separately. Positive donations were confirmed using the RealStar HEV RT-PCR Kit (Altona Roche Diagnostics GmbH, Hamburg, Germany) at the National Reference Laboratory in Berne.

During the same time interval, 625,559 Swiss blood donor control samples collected in seven other Swiss cantons were analyzed in pools of 16 donations in Berne using the HEV RNA assay (95% lower sensitivity limit: 18.6 IU/mL) on a Cobas^®^ 8800 (Roche Diagnostics, Rotkreuz, Switzerland).

The analysis did not include any serological data, as the samples were collected during routine blood donation sessions, and only HEV NAT testing was carried out.

### 2.5. Statistical Analyses

The primary outcome of the study was the difference in global IgG prevalence in the two periods studied. Following the introduction of the HEV NAT testing, we also investigated the HEV incidence in southern Switzerland and compared it with that of representative Swiss blood donor controls originating from seven Swiss cantons (Bern, Basel Stadt, Basel Land, Geneva, Wallis, Sankt Gallen, and Vaud).

Continuous data (age) are presented as mean and corresponding 95% confidence intervals (95% CI), median, minimum, and maximum. Frequency data are presented as counts and percentages, and pairwise associations between groups were computed using contingency tables and carrying out chi-square or Fisher’s exact tests, as appropriate. We used a t-test to assess any difference in age across samples and two-sample tests for proportions between samples or geographic regions. No confirmatory tests were carried out across districts or areas, as the sample sizes were too small to obtain reliable estimates, and only 95% CI is presented for age differences with regard to these data.

A logistic regression (model 1) with gender, age class (18–30, 31–45, 46–65, 66+ years), sampling period, and areas as independent variables was used to assess the dependency of IgG or IgM seroprevalence from these variables. We decided to use a categorical binned variable for age to better evaluate in which age range people are more likely to test positive for anti-HEV IgG or IgM. A second logistic regression (model 2) with gender, age, sampling period, and areas as independent variables was used to verify that no loss of information was caused by the use of age classes. Margins (calculated from predictions of the previously fit model 2 at fixed values of the gender covariates and integrating over the remaining covariates) were computed using age (raw data) and sex as covariates, and the resulting data were plotted (Stata routine: marginsplot). The statistical significance level (α) was set at 0.05.

Data originating from HEV NAT testing were analyzed only descriptively and summarized by donation periods. The percentage of positive tests in southern Switzerland (cases) was compared with that of the aggregate donations from the seven cantons Bern, Basel Stadt, Basel Land, Geneva, Wallis, St. Gallen, and Vaud (control samples) with tests on the equality of proportions using large-sample statistics, with the cases proportion as the hypothesized population. Odds ratios were computed using the southern Switzerland data as a case cohort.

Stata Version 17 (StataCorp LCC, College Station, TX, USA) was used for all statistical analyses and for the preparation of graphical displays.

## 3. Results

### 3.1. Demographics and Geographic Distribution of the Blood Donors

The sex and age characteristics and the distribution between regions and districts of the blood donors tested for HEV IgG and IgM are summarized in Table 1 and Table S1. No statistically significant differences between the 2012–2013 and 2017–2019 populations could be detected with regard to sex, age, or geographic distribution.

### 3.2. HEV IgG and IgM Seroprevalence and Regional Distribution

The overall HEV IgG prevalence in southern Switzerland was 32.8% from 2012 to 2013 and 31.1% from 2017 to 2019 (Table 2), a difference not statistically significant (*p* = 0.337). The value was 27.5% from 2012 to 2013 and 26.8% from 2018 to 2019 in female donors, as opposed to 35.1% and 33% in male donors. The difference in frequencies between male and female gender is statistically but not clinically significant (2012–2013: *p* = 0.007; 2017–2019: *p* = 0.018). No confirmatory tests were carried out for districts or regions, as the size of the samples was too small for these evaluations.

**Figure 1:**
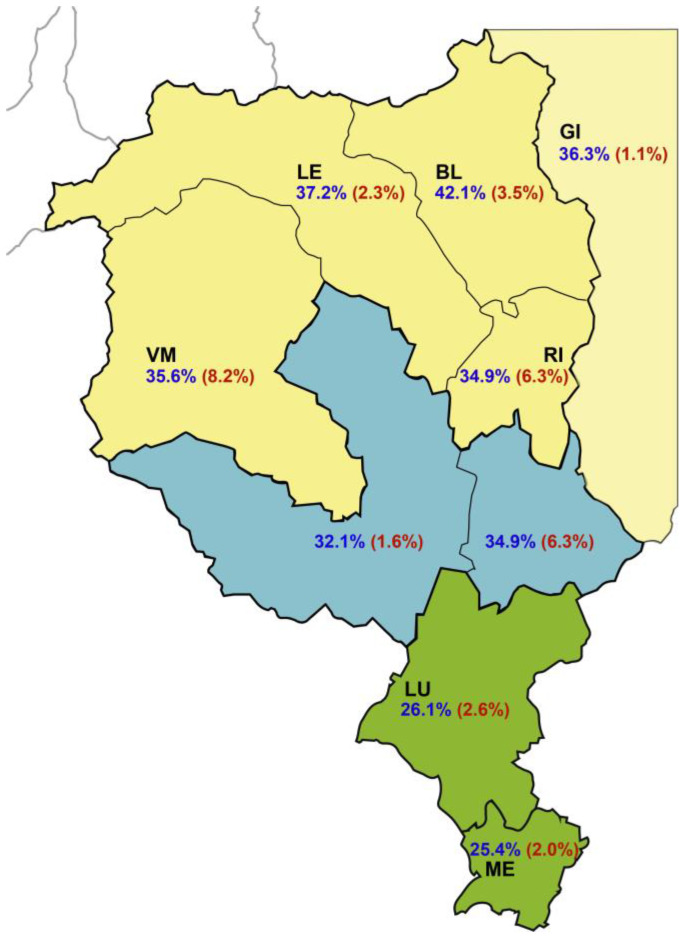
Regional and district distribution of the HEV IgG (navy color) and IgM (red, in parentheses) seroprevalence in blood donors, 2017–2019. BE, Bellinzona; BL, Blenio; GI, Grigioni Italiano; LE, Leventina; LO, Locarno; LU, Lugano; ME, Mendrisio; RI, Riviera; VM, Vallemaggia. Green fill: south; blue fill: center; yellow fill: north; Grigioni Italiano: light yellow.

The HEV prevalence in the different districts and areas is presented in Table 2, Table S2, and Figure 1. For IgM, the overall prevalence was 2% from 2012 to 2013 and 2.8% from 2017 to 2019 (Table 2). In females, it was 1.8% from 2012 to 2013 and 2.3% from 2017 to 2019, as opposed to 2.1% and 3% in males, but in both instances, the difference was not statistically significant.

A gradient in IgG from the south to the north can be seen, and the overall differences among the three areas are statistically significant in both sampling periods, except for the south and center during the sampling period 2012–2013. No statistically significant differences among areas could be detected for the IgM seroprevalence.

**Figure 2:**
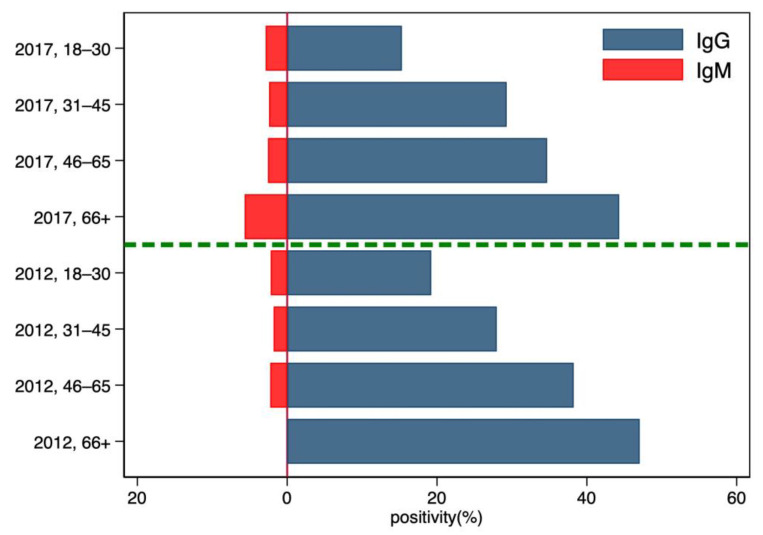
Distribution of the HEV IgG and IgM seroprevalence in blood donors according to their age in the two sampling periods 2012–2013 and 2017–2019.

With regards to the age of the donors (Figure 2), we observed an increase in IgG seroprevalence with age, whereas the IgM seroprevalence is roughly constant over time.

**Figure 3:**
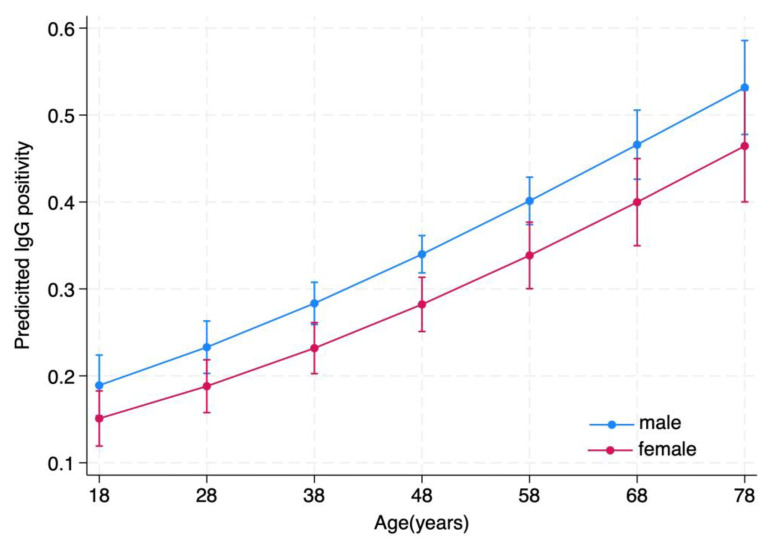
Predicted changes of anti-HEV IgG in relationship to age (marginsplot). Outcome of the logistic regression using gender, age, sampling period, and areas as independent variables.

The logistic regression (model 1; Table 3) indicates that age and donor provenance influence the occurrence of a positive IgG seroprevalence, with donors older than 66 years being almost four times more likely to be seropositive than 18–30-year-old donors and males apparently more at risk than females (Figure 3). With regards to age, model 2 using age (and not age category) as a covariate produced almost identical results (Table S3). Predicted changes in IgG prevalence showed a positive, almost linear relationship with age (Figure 3). Donors from the more rural north area are 1.7 times more likely to develop a positive IgG serology. Interactions terms were not statistically significant. No statistically significant dependencies were evident from the same analysis carried out for IgM, although a statistically non-significant difference could be observed for the north area as compared with the south and the center (OR for north: 1.62; 95% CI: 0.93–2.82, *p* = 0.087).

### 3.3. Change in IgG Positivity from 2012–2013 to 2017–2019 in the Donors Included in Both Groups

A total of 303 donors were included in both 2012–2013 and 2017–2019 samples. In this population, the IgG status did not change (either remained positive or negative depending on the 2012–2013 status) for 82% of the donors. A loss of positivity was observed only in approx. 6% of them, whereas IgG seropositivity was developed by 11% of the donors (Table 4).

### 3.4. HEV NAT Incidence after 2018

**Figure 4:**
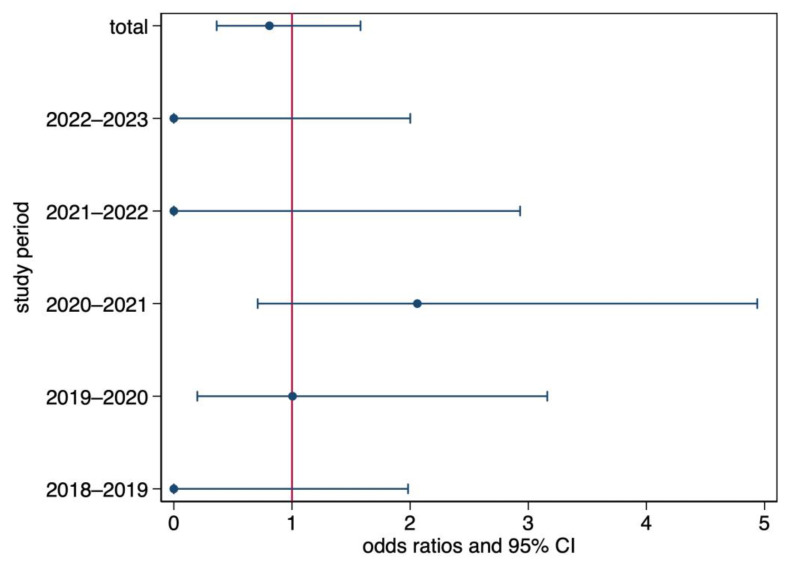
Odds ratios (point estimates: dots) and their 95% confidence intervals (lines) of a positive test in the southern Switzerland sample as compared to the Swiss control.

Since the introduction in 2018 of the mandatory HEV NAT testing of blood donations, 49,345 donations have been tested in southern Switzerland and 625,559 in the seven cantons managed by the IRB (Table 5). In southern Switzerland, nine (0.02%) HEV NAT-positive cases were confirmed by the National Reference Laboratory. Four of them occurred in February 2021, during a countrywide outbreak of HEV cases, with an increase in NAT-positive donors at the national level, whereas the other five were detected before the outbreak. Of the four cases reported in February 2021 in southern Switzerland, only one could be phylogenetically assigned to the outbreak, and two could not be reliably identified because the viral load was too low. Except for the periods 2019–2020 (no difference) and 2020–2021 (higher incidence in southern Switzerland), the proportion of positive cases in southern Switzerland was smaller than in the control Swiss population (Table 5). All pairwise differences were statistically significant (*p* < 0.001), but the multivariate model detected no statistically significant differences (Figure 4).

## 4. Discussion

Our data confirm that blood donors in southern Switzerland have the highest overall IgG seroprevalence in Switzerland (31.1%) [23] and that the IgG seroprevalence did not change between 2012–2013 and 2017–2019, although an important increase in the number of clinical HEV cases was observed during this period [20].

A study performed in Switzerland between 2014 and 2016 described an overall HEV IgG seroprevalence in blood donors of 20.4%, with a minimum of 12.8% in Geneva and a maximum of 33.6% in southern Switzerland [23]. We tried to confirm these data by investigating the HEV IgG prevalence in a large number (n = 1447) of blood donors during the period 2017–2019 and also analyzed 1283 samples of donors collected from 2012 to 2013. Surprisingly, the HEV prevalence and distribution in the territory remained unchanged. This is in contrast with observations made during the same period in other Swiss regions, for example, in Lausanne (Canton Vaud), where the IgG prevalence increased from 4.9% to 22.2% from 2011 to 2017 [23,34]. Caution, however, is needed in the interpretation of these results because the two studies relied on two different immunological assays, the Wantai ELISA [23] and the less sensitive MP Diagnostics HEV ELISA [34].

Few studies document the course of the HEV IgG through the years. In our study, we were able to analyze a subgroup of 303 donors who were included in both periods (2012–2013 and 2017–2019). Of these, 35 (11.5%) acquired IgG positivity during the 6-year interval between the two observations, thus confirming the increase observed with increasing age in our and other studies [23,35,36]. Interestingly, 18 (5.9%) of the participants in our study lost IgG positivity after the 7-year follow-up. This strengthens the hypothesis suggested by previous studies that a small proportion of HEV IgG disappears from the blood over time, probably some years [35,37,38].

IgM rates from 2017 to 2019 remained almost unchanged in all districts or areas compared to the 2012–2013 period. According to published studies, HEV IgM persists on average for 5–6 months, although a few cases of HEV IgM persistence for 8–12 months have also been reported [15,37,39,40,41]. Therefore, no HEV infections acquired in 2013 should have persisted 6 years later and caused false positives.

We also compared NAT-positive cases in southern Switzerland with those observed in the rest of the country and did not find any statistically significant difference. Our data, therefore, suggest that food chain control measures are effective in reducing cases of acute hepatitis E with or without extrahepatic complications [20], but sudden outbreaks such as that observed in Switzerland in February 2021 [42] are still possible.

Our study has shown that men and elderly donors living in rural areas are at higher risk of being positive for HEV IgG. The difference in seroprevalence between northern rural and southern urban areas may be explained by the large number of farmers in rural areas in close contact with animals, in particular pigs. HEV infection, therefore, could be the logical consequence of zoonotic infections [43]. In addition, differences in eating habits and grocery patterns may explain the different seroprevalence: The northern rural populations probably eat more regional food, such as the local butcher’s made “mortadella” with raw pig liver, which is difficult to find in large grocery stores in urban areas. Young people in urban areas usually eat food acquired in supermarkets, delivering food from all over the country. Indeed, two Swiss studies performed between 2016 and 2018, one in pig farms [44] and the other in ready-to-eat meat products [32], showed different HEV contamination rates of 50% and 5–19%, respectively. On the other hand, the higher numbers of IgG in elderly people might also be explained by the accumulation of asymptomatic infection during life.

Our study has some limitations. We compared seroprevalences between two different, unpaired donor populations to address the primary endpoint set in the protocol (difference in global IgG prevalence during the two periods studied). Data from Table 1, however, confirm that no major statistically significant differences in the demographics of the donors could have influenced the risk of IgG positivity. In addition, using the same serological assay in the two populations, overall seroprevalence and its distribution across the areas of provenience did not change. Case–control matching between 2012–2013 and 2017–2019 samples for sex, age, and district of provenience would have been an alternative approach, but logistically, this would have been too complex; thus, we decided to obviate this limitation, at least partly, by selecting cases that eventually could yield similar distributions with respect to the demographics used in the analysis. A within-donor design would have been possible only by collecting additional data, such as possible changes in health status, domicile, and food preferences, in addition to sex and age. This choice would have implied a prospective study design, almost impossible for an epidemiological study with blood donors. The lack of a within-donor comparison, however, is a limitation only for secondary endpoints that analyze individual changes. This has been partly addressed by the seroprevalence changes presented in Table 4. We used logistic regression for models 1 and 2, but we decided against using any more specialized Generalized Linear Model for this paired analysis because too many additional, unknown random factors could not be accounted for in the model using the data available.

An additional limitation is given by the use of pooled HEV NAT testing, which could have led to a dilution of the HEV RNA and to false negative results, thus underestimating HEV prevalence. The absence of NAT-positive samples in southern Switzerland in the period 2021–2023 could be a result of this situation. The design of the study also does not allow detection of all HEV cases. We included in our study only blood donors, less than 10% of the whole population, to evaluate, more specifically, the risk of HEV transmission related to infected, asymptomatic donors through blood products. Prevalence and incidence rates from patients referred to a gastroenterology practice would probably be higher, but this population would not be representative of a blood donor population, and asymptomatic cases would not be included. Censoring of sick or acutely infected people leads to an underestimation of the disease burden, but our study was designed to analyze blood donation data. Acute, asymptomatic cases in the blood donor population would have been recorded for the 2017–2019 period, but the retrospective design did not allow for additional data collection in the 2012–2013 period.

We are aware of an HEV vaccine authorized for marketing in China and Pakistan, which could limit the spread of this pathogen and, thus, clinical HEV manifestations [45]. Workers with high occupational exposure to animals such as pigs, wild boars, or deer could certainly benefit from this vaccine [43], and if the HEV burden increases, efforts to have this vaccine approved in additional countries should be made.

In conclusion, the perception of HEV has changed in recent years. For example, in Germany, the number of cases reported to the Robert Koch Institute increased from less than 40 to more than 400 within 10 years [46]. Epidemiological data are useful to better understand the spread of the disease and immediately identify local outbreaks. In fact, analysis of all blood donations collected after the introduction of the mandatory HEV NAT screening has allowed the detection of the 2021 outbreak [42] and shows that cases of HEV viremia still occur in the donor population. In southern Switzerland, food chain control measures were effective in reducing the number of clinically affected HEV patients in the general population, but our long-term data on blood donors show that the infection patterns are more complex and do not vary over time. For this reason, we believe that in addition to the measures introduced to prevent HEV infection from food, testing of blood donations (for example, using HEV NAT) is still required to ensure blood safety, especially for patients at higher risk of transfusion as immunosuppressed or pregnant women, because for them HEV infection represent a life-threatening condition.

## Figures and Tables

**Table 1:** Demographics and regional distribution of the blood donors tested for HEV IgG and IgM. 95% CI: 95% confidence interval of the mean. No statistically significant difference between the two samples was detected for any variable.

	2012–2013 (N = 1283)	2017–2019 (N = 1447)
Overall		
Female		
Count (% of each sample)	392 (30.55)	441 (30.48)
Mean age (95% CI)	43.93 (42.59–45.27)	45.21 (43.95–46.48)
Median age (min–max)	45 (18–71)	46 (18–74)
Male		
Count (% of each sample)	891 (69.45)	1006 (69.52)
Mean age (95% CI)	47.24 (46.46–48.01)	47.74 (46.95–48.52)
Median age (min–max)	48 (18–72)	50 (18–75)
Total		
Mean age (95% CI)	46.24 (45.56–46.92)	46.97 (46.30–47.64)
Median age (min–max)	47 (18–72)	49 (18–75)
By region		
South		
Count (% of each sample)	531 (41.39)	625 (43.19)
Mean age (95% CI)	46.01 (44.96–47.05)	47.41 (46.37–48.44)
Median age (min–max)	48 (18–72)	50 (18–73)
Center		
Count (% of each sample)	305 (23.77)	412 (28.47)
Mean age (95% CI)	47.76 (46.35–49.17)	47.04 (45.76–48.31)
Median age (min–max)	49 (18–71)	48 (18–75)
North		
Count (% of each sample)	447 (34.84)	410 (28.33)
Mean age (95% CI)	45.46 (44.31–46.61)	46.21 (44.99–47.43)
Median age (min–max)	46 (18–70)	48 (18–74)

**Table 2:** IgG and IgM seroprevalence in blood donors (frequency (%)): overall and regional distribution. Percentage values are percentage within each factor-variable level. No statistically significant difference between sampling periods has been detected (IgG: *p* = 0.337; IgM: *p* = 0.21).

	N IgG Pos (%)		N IgM Pos (%)	
	2012–2013	2017–2019	2012–2013	2017–2019
Overall	421 (32.81)	450 (31.10)	26 (2.03)	40 (2.76)
By gender				
Female	108 (27.55)	118 (26.76)	7 (1.79)	10 (2.27)
Male	313 (35.13)	332 (33.00)	19 (2.13)	30 (2.98)
By area				
South	154 (29.00)	162 (25.92)	9 (1.69)	15 (2.40)
Center	93 (30.49)	136 (33.01)	5 (1.64)	9 (2.18)
North	174 (38.93)	152 (37.07)	12 (2.68)	16 (3.90)

**Table 3:** IgG seroprevalence in blood donors: results of the logistic regression analysis using gender, age category, sampling period, and areas as independent variables. Interactions terms were not statistically significant. OR: odds ratios; SE: standard error; *p*: statistical significance; 95% CI: 95% confidence intervals of the OR.

	OR	SE	*p*	95% CI
Gender				
Male	Reference			
Female	0.68	0.193	0.171	0.39–1.18
Age category (years)				
18–30	Reference			
31–45	1.86	0.358	0.001	1.27–2.71
46–65	2.59	0.462	<0.001	1.82–3.67
66+	3.87	1.019	<0.001	2.30–6.48
Sampling period				
2012–2013	Reference			
2017–2019	0.922	0.078	0.337	0.78–1.09
Region				
South	Reference			
Center	1.23	0.123	0.047	1.01–1.52
North	1.68	0.165	<0.001	1.39–2.04

**Table 4:** Changes (percent) in IgG results in blood donors, 2012–2018.

IgG Overall	South	Center	North	Total
Unchanged	77 (25.41)	32 (10.56)	141 (46.53)	250 (82.51)
Acquired	12 (3.96)	10 (3.30)	13 (4.29)	35 (11.55)
Loss	7 (2.31)	2 (0.66)	9 (2.97)	18 (5.94)
Total	96 (31.68)	44 (14.52)	163 (53.80)	303 (100.00)

**Table 5:** Results of the HEV NAT tests on donations made between 17 September 2018 and 16 March 2023. In parentheses: proportion (percentage) of positive cases.

	Southern Switzerland			Control (7 Swiss Cantons)		
Period	Positive	Negative	Total	Positive	Negative	Total
2018–2019	0 (–)	5394	5394	26 (0.035)	72,381	72,407
2019–2020	3 (0.027)	10,885	10,888	39 (0.027)	142,156	142,195
2020–2021	6 (0.053)	11,224	11,230	36 (0.026)	138,800	138,836
2021–2022	0 (–)	10,592	10,592	17 (0.012)	137,390	137,407
2022–2023	0 (–)	11,241	11,241	23 (0.017)	134,691	134,714
Total	9 (0.018)	49,336	49,345	141 (0.022)	625,418	625,559

## Data Availability

The datasets generated and/or analyzed during the current study are available from the corresponding author upon reasonable request.

## Supplementary Materials

The following supporting information can be downloaded at: https://www.mdpi.com/article/10.3390/microorganisms11102375/s1, Fontana_Supplementary_Material_20230816.docx. Table S1. Demographics and distribution of the blood donors tested for HEV IgG and IgM by the districts studied. 95% CI: 95% confidence interval of the mean; Table S2. IgG and IgM seroprevalence in blood donors [frequency (%)]: overall and split by districts; Table S3. IgG seroprevalence in blood donors: Results of the logistic regression analysis using gender, age category, sampling period, and areas as independent variables.
