# Supplementary material for: Epidemiology of HEV Infection in Blood Donors in Southern Switzerland
Source: Microorganisms. 2023 Sep 22;11(10):2375. doi: 10.3390/microorganisms11102375 (PMC10609445; doi:10.3390/microorganisms11102375)
Supplement: Supplementary file 1 [file microorganisms-11-02375-s001.zip › microorganisms-2537121-supplementary.pdf]

Supplementary Material to Fontana et al.

Table S1. Demographics and distribution of the blood donors tested for HEV IgG and IgM by the districts studied. 95% CI: 95% confidence interval of the mean.

	2012-2013 (N=1,283)	2017-2019 (N=1,447)	p-value
**By district**			
Bellinzona			
count (% of each sample)	173 (13.48)	222 (15.34)	
mean age (95% CI)	47.65 (45.81–49.5)	47.26 (45.54–48.98)	
median age (min–max)	49 (19–71)	48 (21–75)	
Blenio			
count (% of each sample)	77 (6.00)	57 (3.94)	
mean age (95% CI)	45.97 (43.51–48.43)	47.16 (44.08–50.24)	
median age (min–max)	45 (23–70)	49 (19–71)	
Leventina			
count (% of each sample)	126 (9.82)	129 (8.91)	
mean age (95% CI)	48.63 (46.46–50.8)	49.47 (47.34–51.6)	
median age (min–max)	49.5 (19–68)	51 (18–71)	
Locarno			
count (% of each sample)	132 (10.29)	190 (13.13)	
mean age (95% CI)	47.90 (45.69–50.11)	46.78 (44.87–48.7)	
median age (min–max)	50 (18–71)	49 (18–73)	
Lugano			
count (% of each sample)	334 (26.03)	428 (29.58)	
mean age (95% CI)	45.62 (44.32–46.93)	47.59 (46.35–48.83)	
median age (min–max)	47 (18–72)	51 (18–73)	
Mendrisio			
count (% of each sample)	197 (15.35)	197 (13.61)	
mean age (95% CI)	46.68 (44.92–48.43)	47.02 (45.13–48.91)	
median age (min–max)	48 (19–72)	50 (18–73)	
Riviera			
count (% of each sample)	78 (6.08)	63 (4.35)	
mean age (95% CI)	44.96 (42.01–47.91)	41.24 (37.84–44.63)	
median age (min–max)	45.5 (19–68)	43 (19–66)	
Vallemaggia			
count (% of each sample)	73 (5.69)	73 (5.04)	
mean age (95% CI)	39.10 (36.13–42.07)	43.75 (40.66–46.85)	
median age (min–max)	40 (19–67)	46 (19–74)	
Grigioni Italiano			
count (% of each sample)	93 (7.25)	88 (6.08)	
mean age (95% CI)	46.17 (43.87–48.48)	46.43 (44.08–48.78)	
median age (min–max)	47 (18–66)	47.5 (21–67)	

Table S2. IgG and IgM seroprevalence in blood donors [frequency (%)]: overall and split by districts.

	N IgG pos (%)			N IgM pos (%)	
	2012-2013	2017-2019		2012-2013	2017-2019
By district					
Bellinzona	61 (35.26)	75 (33.78)		4 (2.31)	6 (2.70)
Blenio	23 (29.87)	24 (42.11)		3 (3.90)	2 (3.51)
Leventina	55 (43.65)	48 (37.21)		3 (2.38)	3 (2.33)
Locarno	32 (24.24)	61 (32.11)		1 (0.76)	3 (1.58)
Lugano	99 (29.64)	112 (26.17)		5 (1.50)	11 (2.57)
Mendrisio	55 (27.92)	50 (25.38)		4 (2.03)	4 (2.03)
Riviera	29 (37.18)	22 (34.92)		1 (1.28)	4 (6.35)
Vallemaggia	23 (31.51)	26 (35.62)		4 (5.48)	6 (8.22)
Grigioni Italiano	44 (47.31)	32 (36.36)		1 (1.08)	1 (1.14)

Table S3. IgG seroprevalence in blood donors: Results of the logistic regression analysis using gender, age category, sampling period, and areas as independent variables. Interactions terms were not statistically significant. OR: Odds Ratios; SE: standard error; p: statistical significance; 95% CI: 95% confidence intervals of the OR.

	OR	SD	p	95% CI
gender				
male	reference			
female	0.76	1.19	0.846	0.02–18.06
age (years)				
18	reference			
>18	1.02	0.03	0.000	1.02–1.03
sampling period				
2012-2013	reference			
2017-2019	0.926	0.078	0.362	0.78–1.09
region				
South	reference			
Centre	1.23	0.129	0.052	0.99–1.50
North	1.70	0.167	<0.001	1.40–2.06
